# Efficacy of supplementing *Aspergillus awamori* in enhancing growth performance, gut microbiota, digestibility, immunity, and antioxidant activity of heat-stressed broiler chickens fed diets containing olive pulp

**DOI:** 10.1186/s12917-024-04050-7

**Published:** 2024-05-17

**Authors:** Abdel-Moneim Eid Abdel-Moneim, Raafat S. Khidr, Aml M. M. Badran, Shimaa A. Amin, Faisal B. Badri, Ghada G. Gad, Hany A. Thabet, Ahmed M. Elbaz

**Affiliations:** 1 Biological Applications Department, Nuclear Research Center, Egyptian Atomic Energy Authority, Cairo, 13759 Egypt; 2 Desert Research Center, Mataria, Cairo, Cairo Egypt; 3 Poultry Breeding Department, Animal Production Research Institute, Agricultural Research Center, Ministry of Agriculture, Dokki, Giza, Giza Egypt; 4 Microbiology Department, Faculty of Agriculture, Ain Shams University, Cairo, Egypt; 5 Poultry Production Department, Faculty of Agriculture, Ain Shams University, Cairo, Egypt

**Keywords:** *Aspergillus Awamori*, Heat stress, Olive pulp, Growth, Gut microbiota, Antioxidant status

## Abstract

**Background:**

Gut microbes play a significant role in digestion, developing immunity, and intestinal health. Therefore, direct-fed microbials are used to modify gut microbiota, maintain a healthy digestive system, enhance immunity, and promote the broilers’ performance. In addition, it has a role in improving the utilization of unconventional feed ingredients (olive pulp, OP). This study provides the potential role of *Aspergillus awamori* in enhancing gut microbial content, nutrient utilization, growth performance, and antioxidative status in heat-stressed broiler chickens fed diets containing olive pulp.

**Methods:**

Three hundred chicks (Ross 308; one day old) were divided into four treatment groups (75 chick/ group) randomly, as follows; CON: chicks fed a basal diet based on corn and soybean meal, OP_10_: chicks fed a diet containing 10% OP, OA1: chicks fed a diet containing OP with *A. awamori* at 100 mg per kg, OA2: chicks fed a diet containing OP with *A. awamori* at 200 mg per kg.

**Results:**

Adding *A. awamori* to the broiler diet that contains OP had a positive effect on productive performance via enhancing nutrition digestibility, body weight gain, feed conversion ratio, and carcass characteristics. *A. awamori* supplementation had a positive impact on immune responses by increasing serum immunoglobulin G and the relative weight of bursa of Fabricius (*P* < 0.05) compared to the other groups. Chickens fed *A. awamori* showed a noticeable improvement in the oxidative status through the increase in the level of serum superoxide dismutase, and glutathione peroxidase, and the decrease in the level of malondialdehyde. Feeding *A. awamori* also modified the intestinal microbial content by increasing the population of *Lactobacillus* (*P* < 0.05).

**Conclusions:**

Our study indicated that adding 200 mg *A. awamori* reduced the negative effect of heat stress by modifying the microbial content of the intestine, immune response, and enhancing feed utilization, thus improving broiler performance, as well as, improving the nutritional value of the olive pulp. Therefore, adding *A. awamori* to the OP diet can be effectively used in heat-stressed broiler diets.

## Background

Poultry products are a cheap protein source (egg and meat) compared to other protein sources (fish and livestock) to provide human protein needs, especially in developing countries. The increased demand for poultry meat led to the continuous development of the breeding house systems, feeding and expanding its upbringing. However, many obstacles affect the sustainability of the poultry industry, some of the most important obstacles are environmental changes and nutrition. Egypt, as one of the developing countries, is suffering from a shortage of conventional feedstuffs (corn and soybean). The recent economic crisis due to Covid-19 as well as other economic problems negatively impacted the poultry industry. Which made the nutritionist search for the use of some agricultural waste or oil extraction residues as alternatives to corn and soybean meal [1–3]. Despite the unquestionable feeding value of both corn and soybean meal, a partial replacement had to be done to reduce feed costs without affecting the performance and health of the bird. In our current study, olive oil extraction residues were selected, especially since Mediterranean countries are characterized by high production of olive oil, which produces large amounts of waste (cake and residual oil) [4].

Olive pulp (OP) is one of the most olive oil extraction products, especially since it is rich in fatty acids (oleic, linoleic, and linolenic), crude protein, calcium, and copper [5, 6], as well as, some biologically active compounds (polyphenol) that have an antioxidant, anti-inflammatory and antibacterial properties [7]. However, OP has low nutritional value due to its low energy, and indigestible proteins, in addition to containing high fiber content and lignin [4]. It was necessary to use some feed supplements in chicken diets containing these by-products (such as OP) to reduce antinutritional factors, thereby reducing their negative influence on chicken production performance. Furthermore, the poultry industry faces the risk of heat stress resulting from environmental changes with the emergence of global warming, which led to significant economic losses in the poultry industry [8, 9]. Exposure to heat stress leads the outbreak of contagious diseases and threatens the growth, nutrient uptake, digestibility, and physiological functions of the host [8]. Moreover, heat stress suppresses the innate immune response and induces immune disorders by altering the organs’ immune functions [9]. Heat stress also induces oxidative stress by damaging the membrane of immune cells, leading to apoptosis and increased intestinal barrier permeability and consequently translocation of toxic agents into the body [10]. Consequently, it is important to implement mitigation strategies for environmental heat stressors, such as using some feed additives, including vitamins, oils, organic acids, probiotics, etc [9, 11].

Direct-fed microbials are live microorganisms that confer health benefits on the host. Modification of the gut microbiota is the most important mechanism and plays an important role in maintaining homeostasis and the physiology of the bird, in addition to its direct contribution to bird health and productivity. *Aspergillus awamori* is a fungus that has been used in food for a long time in Japan. The Food and Drug Administration of America announced that it is safe to use [12]. It also plays an important role as much as probiotics in improving the gut health of the host (bird) by changing the microbial content [11]. Furthermore, it can produce some enzymes (e.g., α-amylase, glucoamylase, and protease) that improve the utilization of protein and carbohydrates [13]. Moreover, it produces citric acid which enhances growth by acidifying the gastrointestinal contents, reducing pathogenic colonization, and improving nutrient digestibility [12]. Thus improving immune and oxidative status [14]. In this study, *A. awamori* as a probiotic was selected, for its role in modifying the microbial content of the intestine and improving the utilization of nutrients during heat stress [10], in addition, to enhancing the nutritional value of OP [7]. We assumed that the addition of *A. awamori* will improve the nutritional value of OP by improving nutrient utilization, as well as, reducing the harmful effect of heat stress on chicks. Therefore, different levels of *A. awamori* were added to broiler chicken diets that included OP and evaluated its impact on growth performance, carcass characteristics, serum metabolism, antioxidant status, and gut microbiota diversity in broilers exposed to heat stress.

## Materials and methods

### Birds, diets, and management practices

A total of 300 broiler chicks (Ross 308) at 1-day-old were obtained from a commercial hatchery (Wadi Poultry Company). Broiler chicks were randomly allocated into four dietary treatments, 5 replicates per group (15 chicks per replicate), as follows: 1: chicks fed a basal diet of corn and soybean meal (CON), 2: chicks fed a diet containing 10% OP to replace part of the corn-soybean meal (OP_10_), 3: chicks fed a diet containing 10% OP with *A. awamori* at 100 mg per kg diet (OA1), and 4: chicks fed a diet containing 10% OP with *A. awamori* at 200 mg per kg diet (OA2). The chemical composition of olive pulp [15] was 9.4% crude protein, and 23.8% crude fiber, as shown in Table 1. OP was obtained from a local olive oil manufacturer, then it was dried and processed. *A. awamori* (25 × 10^4^ cells per g) was provided in powder form from Biogenkoji Research Institute, Kirishima, Japan. The starter diet (0–21 days) and grower diet (21–35 days) were formulated according to NRC [16], as shown in Table 2. The olive meal energy in the diet was calculated according to Elbaz et al. [17]. Chicks feed and water were provided ad libitum. The room temperature was maintained at 32.5 °C for the first two days and then gradually reduced by 3 °C weekly until reaching 23 °C, then maintained for the rest of the experiment. Starting on the 6th-day, birds were exposed to heat at 33 °C for 4 hours/daily for 4 days a week until the end of the experiment (To simulate summer conditions in Egypt) with continuous light in all experiment stages.

**Table 1:** Chemical composition of olive pulp used in the experiment

Item	DM (%)	CP (%)	EE (%)	Ash (%)	CFA (%)	Ca (%)	*P* (%)
Olive pulp	91.5	9.4	23.8	7.9	12.6	0.5	0.1

DM: Dry matter; CF: crude fiber; CP: crude protein; EE: ether extract; Ca: calcium; and P: Phosphorus

**Table 2:** Composition and calculated analysis of experimental diets fed to broiler chickens

Ingredient (%)	Starter	1–21 days	Grower	22–35 days
	CON	OP	CON	OP
Olive pulp	0.00	10.00	0.00	10.00
Yellow corn	56.7	44.25	61.4	49.8
Soybean meal	32.0	33.40	26.53	26.6
Corn gluten meal	5.00	5.00	5.00	5.00
Sunflower oil	2.13	3.38	3.24	4.77
Di-Calcium Phosphate	2.10	2.10	1.90	1.90
Calcium carbonate	1.27	1.12	1.06	1.02
Mineral mix^1^	0.15	0.15	0.15	0.15
Vitamin mix^2^	0.15	0.15	0.15	0.15
Salt	0.20	0.20	0.20	0.20
DL-Methionine	0.20	0.15	0.20	0.20
Hcl-Lysine	0.00	0.00	0.07	0.11
Sodium bicarbonate	0.10	0.10	0.10	0.10
Calculated analysis				
Crude protein (%)	22	22	20	20
ME (Kcal / kg)^3^	3000	3000	3150	3150
Crude fiber (%)	3.59	5.06	3.37	5.44
Calcium (%)	1.05	1.06	0.92	0.91
Av. phosphorus (%)	0.51	0.51	0.46	0.46

^1^Supplied per kilogram of diet: 150 mg Choline, 48 mg Ca, 3.18 mg P, 100 mg Mn,, 0.25 mg Co, 1.5 mg Iodine, 50 mg Fe, 80 mg Zn, 10 mg Cu.^2^Supplied per kilogram of diet: 1400 IU vitamin A, 3000 IU Vitamin D3, 3 mg Vitamin B6, 50 mg vitamin E, 4 mg vitamin K, 20 mg Pantothenic acid, 6 mg Vitamin B12, 60 mg Niacin, 0.20 mg folic acid.^3^ME=Metabolizable energy

### Sampling collection

At the age of 35 days, 20 birds were slaughtered (Koechner Euthanizing Device), 5 birds from each group, to obtain carcass characteristics, blood samples, and microbial count samples from the cecum (2 g of cecum contents) and kept at 10 °C until analyzed, in addition, the contents of the cecum (about 2–3 g) were emptied to estimate the activity of the digestive system enzymes. Blood samples were drawn from the jugular vein right, samples were centrifuged at 3000 × g for 15 min, and then serum was stored at − 10 °C until analyzed. Five birds were separated from each group and placed in individual cages to conduct a digestion experiment after starving the birds for 8 h to empty the digestive tract, after three days of collecting and weighing excreta and feed, they were dried and stored until to evaluate the nutrient digestibility.

### Performance parameter

Live body weight (LBW), feed intake (FI), and health status of broiler chickens from the start of the experiment until 35 days of age were recorded. Feed conversion ratio (FCR), body weight gain (BWG), and survival rate were calculated. After the slaughtering process, the digestive tract and internal organs such as the pancreas, liver, gizzard, bursa of Fabricius, spleen, and thymus were separated and weighed on 0.001 g digital scale, in addition to relative weights of the thigh, breast, and abdominal fat, and dressing percentage was calculated. The relative weight of each organ was calculated as follows: Relative weight = (organ weight/live body weight) x 100.

### Chemical analysis

Dry matter (AOAC #, 934.01), crude protein (AOAC #, 954.01), ether extract (AOAC #, 920.39), and crude fiber (AOAC #, 962.09) were determined according to the method of the Association of Official Analytical Chemists [18], to estimate nutrient digestibility. To estimate the effect of treatments on the activity of digestive enzymes, cecum digesta was collected from the slaughtered birds and diluted 4 and 10 times with phosphate-saline buffer followed by centrifugation at 8,000× g for 20 min at 4 °C. The supernatant was collected to lipase, protease, and amylase activity was analyzed using the method described by Najafi et al. [19], Lynn and Clevette-Radford [20], and Elbaz et al. [21] respectively.

### Biochemical indices

Serum cholesterol, triglycerides, HDL, LDL, glucose, total protein, albumin, aspartate aminotransferase (AST), alanine aminotransferase (ALT), and alkaline phosphatase (ALP) were measured by an automatic biochemical analyzer (CX9, Beckman). Chick serum-specific antibodies (immunoglobulins) such as IgA, IgG, and IgM concentrations were determined in diluted samples (1:100) by enzyme-linked immunosorbent assay ELISA) using microtiter plates (NuncImmunoplate 96-well, cat. no. 446,612) as per manufacturer’s ELISA quantitation kits (Bethyl Laboratories Inc., Montgomery, TX, USA). Serum malondialdehyde (MDA), superoxide dismutase (SOD), and glutathione peroxidase (GPx) levels were determined according to the instruction of the commercial kits of Nanjing Jincheng Bioengineering Institute (China).

### Cecum microbial enumeration

For microbial estimation, the cecum contents were squeezed into sterilized stomacher bags at 35 days of age. Five fresh samples (3 g/sample) from each group were diluted and plated onto MacConkey agar, Rogosa, deMan, Sharpe agar, and plate count agar to enumerate *Lactobacillus, coliforms, Escherichia coli*, and *Enterococcus* respectively, and the number of microorganisms was converted to log^10^ [22].

### Statistical analysis

Collected data were statistically analyzed using the one-way analysis of variance (ANOVA) of the statistical analysis software SPSS (v. 20.0; SPSS Inc., Chigaco, IL, USA). Duncan Multiple Range Test [23] was performed to detect differences among treatments at a significance level of 0.05.

## Results

### Growth performance

Table 3 shows the effect of supplementing with A. awamori on the productive performance of broilers fed a diet containing OP under heat stress conditions. Chickens fed 200 mg *A. awamori* (AO2) showed a significant increase (*P* < 0.05) in LBW and BWG compared to chickens fed AO1, OP10, and the control group. However, feed intake (FI) was not affected (*P* < 0.05) among the experimental groups during all trial periods. In addition, the experimental treatments led to a significant change in FCR during all experimental stages. FCR decreased (*P* < 0.05) in chickens received 200 mg *A. awamori* compared to the other experimental groups during 1–21 d and 22–35 days. Overall, FCR significantly decreased in the group fed AO2, followed by the group fed AO1 (*P* < 0.05) compared with the group fed OP10 and the control. Liver, thigh, breast, pancreas, and gizzard relative weights were not statistically affected by OP, A. awamori, or both combined (Table 4). Meanwhile, dressing (%) increased, and abdominal fat (*P* < 0.05) decreased in broilers fed a diet including 200 mg *A. awamori* (OA2) compared with the other groups (*P* < 0.05).

**Table 3:** Growth performance of heat-stressed broilers fed diets containing olive pulp with different levels of *A. awamori* at 35 days

Parameters	CON	OP_10_	OA1	OA2	SEM	*p*-Value
LBW (g)						
IBW	39.52	39.73	39.48	39.65	0.051	0.256
21 days	746^b^	738^b^	749^b^	767^a^	9.263	0.020
35 days	1685^b^	1670^b^	1691^b^	1743^a^	14.09	0.003
BWG (g/bird/day)						
1–21 days	33.5^b^	33.2^b^	33.7^b^	34.5^a^	0.074	0.027
22–35 days	66.8^b^	66.5^b^	67.2^b^	69.7^a^	0.153	0.015
1–35 days	48.1a^b^	47.6^b^	48.4a^b^	49.6^a^	0.086	0.001
FI (g/bird/day)						
1–21 days	45.2	45.1	45.3	45.2	0.218	0.409
22–35 days	136.4	135.4	135.7	136.6	0.627	0.287
1–35 days	81.7	81.3	81.5	81.8	0.106	0.713
FCR (g feed/g gain)						
1–21 days	1.35^ab^	1.36^a^	1.34^ab^	1.32^b^	0.017	0.025
22–35 days	2.04^a^	2.05^a^	2.02^a^	1.96^b^	^0.032^	0.019
1–35 days	1.69^b^	1.72^a^	1.68^b^	1.65^c^	0.004	< 0.001

CON, a basal diet; OP_10_, diet contains 10% olive pulp; OA1, diet contains 10% olive pulp with *A. awamori* at 100 mg; OA2, diet contains 10% olive pulp with *A. awamori* at 200 mg. a–b Means within the same row with different superscripts differ. SEM, standard error of means. IBW, Initial body weight; LBW, live body weight, FI; feed intake, FCR, feed conversion ratio

**Table 4:** Carcass characteristics (%) of heat-stressed broilers fed diets containing olive pulp with different levels of *A. awamori* at 35 days

Parameters	CON	OP_10_	OA1	OA2	SEM	*p*-Value
Dressing	70.54^b^	70.31^b^	71.08^ab^	71.61^a^	2.080	0.027
Breast	24.15	24.09	24.25	24.21	0.029	0.113
Thigh	16.27	16.21	16.30	16.29	0.115	0.095
Gizzard	1.96	2.01	1.99	1.97	0.027	0.207
Abdominal fat	0.66^a^	0.64^a^	0.58^ab^	0.46^b^	0.016	0.016
Pancreas	0.29	0.31	0.30	0.28	0.008	0.088
Liver	2.32	2.29	2.31	2.35	0.013	0.153

CON, a basal diet; OP_10_, diet contains 10% olive pulp; OA1, diet contains 10% olive pulp with *A. awamori* at 100 mg; OA2, diet contains 10% olive pulp with *A. awamori* at 200 mg. a–b Means within the same row with different superscripts differ. SEM, standard error of means. Carcass characteristics are expressed as a percentage of live body weight

### Nutrient digestibility and enzymatic activity

The effect of dietary treatments on nutrient digestibility and digestion enzymes activities of heat-stressed broilers at day 35 are shown in Table 5. Broilers fed *A. awamori* diets at 200 mg/kg diet (OA2) had significantly increased digestibility of crude protein, and crude fiber compared to broilers fed other diets (*P* < 0.05). The digestibility of the dry matter and ether extract was not affected by the experimental supplementation (*P* < 0.05). Broilers fed 200 mg *A. awamori* diets presented significantly higher protease and amylase enzyme activity (*P* < 0.05) compared to the AO1, OP10, and control groups (Table 5). Additionally, the protease activity was significantly higher in broilers fed the diet with 100 mg *A. awamori* (OA1) compared to the OP10 and control groups, while the lipase enzyme activity was not affected by the experimental treatments.

**Table 5:** Nutrient digestibility (%) and enzymatic activity (U/ml) of heat-stressed broilers fed diets containing olive pulp with different levels of *A. awamori* at 35 days

Parameters	CON	OP_10_	OA1	OA2	SEM	*p*-Value
**Nutrient digestibility**						
Dry matter	71.29	71.06	71.31	72.02	0.977	0.083
Ether extract	86.51	87.02	86.64	86.59	0.353	0.405
Crude protein	73.80^b^	72.65^c^	74.47^b^	75.71^a^	0.126	0.021
Crude fiber	67.22^b^	65.95^c^	67.43^b^	68.68^a^	0.069	< 0.001
**Enzymatic activity**						
Protease	11.21^c^	10.96^c^	12.56^b^	14.27^a^	0.504	< 0.001
Amylase	4.07^b^	4.13^b^	4.71a^b^	5.34^a^	0.122	0.013
Lipase	7.35	7.59	7.26	7.41	0.087	0.140

CON, a basal diet; OP_10_, diet contains 10% olive pulp; OA1, diet contains 10% olive pulp with *A. awamori* at 100 mg; OA2, diet contains 10% olive pulp with *A. awamori* at 200 mg. a–b Means within the same row with different superscripts differ. SEM, standard error of means

### Biochemical indices

Biochemical indices were positively influenced by combining *A. awamori* and OP in heat-stressed broiler (Table 6). Triglycerides and cholesterol levels decreased (*P* < 0.05) in chickens fed OA2 compared to other groups under heat stress. LDL level lowered significantly (*P* < 0.05) in the OA2 group than the other groups. HDL level increased significantly in chickens fed 200 mg *A. awamori* (*P* < 0.05) compared with other experimental groups. However, there was a slight increase in the level of HDL in chickens fed 100 mg *A. awamori* (AO1) compared to OP10 and control group. Total protein, albumin, aspartate aminotransferase (AST), alanine aminotransferase (ALT), and alkaline phosphatase (ALP) concentrations were not changed among the experimental groups. Oxidative enzyme activity greatly differed among the experimental groups (Table 7). GPx and SOD levels increased (*P* < 0.05) in broiler fed OA2 compared to OA1, OP10, and control. Moreover, MDA decreased slightly (*P* < 0.05) in broiler fed OA2 compared to other groups. Furthermore, the SOD level was higher slightly in the chickens fed on OP and AO1 (*P* < 0.05) than in chickens fed the control diet, while the highest level was SOD in the group fed with OA2.

**Table 6:** Biochemical indices of heat-stressed broilers fed diets containing olive pulp with different levels of *A. awamori* at 35 days

Parameters	CON	OP_10_	OA1	OA2	SEM	*p*-Value
Triglycerides	217^a^	201^b^	196^b^	182^c^	2.047	0.001
Cholesterol	231^a^	224^a^	227^a^	216^b^	1.009	0.040
LDL	144.2^ab^	152.7^a^	141.5^ab^	127.8^bc^	0.871	< 0.001
HDL	56.8^b^	51.5^b^	61.9^ab^	70.2^a^	0.095	0.031
Glucose	229	231	225	213	0.469	0.243
Total protein	3.86	3.57	3.68	3.97	0.027	0.116
Albumin	2.07	2.11	2.03	2.19	0.133	0.052

CON, a basal diet; OP_10_, diet contains 10% olive pulp; OA1, diet contains 10% olive pulp with *A. awamori* at 100 mg; OA2, diet contains 10% olive pulp with *A. awamori* at 200 mg. LDL, low-density lipoprotein; HDL, high-density lipoprotein; a–b Means within the same row with different superscripts differ. SEM, standard error of means

**Table 7:** Liver and antioxidant enzymes of heat-stressed broilers fed diets containing olive pulp with different levels of *A. awamori* at 35 days

Parameters	CON	OP_10_	OA1	OA2	SEM	*p*-Value
**Liver enzymes**						
AST (U/L)	192	203	195	193	3.029	0.137
ALT (U/L)	24.7	23.4	23.9	24.3	0.054	0.085
ALP (U/L)	22.1	21.7	22.3	21.5	0.021	0.206
**Antioxidant enzymes**						
SOD (U/ ml)	1.125^c^	1.306^bc^	1.347^b^	1.411^a^	0.008	< 0.001
MDA (nmol/ ml)	1.594^a^	1.604^a^	1.518^ab^	1.465^b^	0.006	0.020
GPx (U/ ml)	19.61^b^	18.80^b^	20.03^b^	23.94^a^	0.110	0.005

CON, a basal diet; OP_10_, diet contains 10% olive pulp; OA1, diet contains 10% olive pulp with *A. awamori* at 100 mg; OA2, diet contains 10% olive pulp with *A. awamori* at 200 mg. a–b Means within the same row with different superscripts differ. SEM, standard error of means

### Immune response

The effect of dietary treatments on immune responses such as immunoglobulins and lymphoid organs of heat-stressed broilers are shown in Table 8. Broilers fed *A. awamori* diets at 200 mg/kg diet (OA2) had significantly higher levels of IgG and relative weight bursa of Fabricius compared to broilers fed OA1, OP_10_, and control diet (*p* < 0.05). The relative weight of the thymus and spleen was not affected by *A. awamori* supplementation (*p* < 0.05). Additionally, the IgM and IgA levels were not affected (*p* < 0.05) by the experimental treatments.

**Table 8:** Immune responses (immune organs (%) and immunoglobulin (mg/dl)) of heat-stressed broilers fed diets containing olive pulp with different levels of *A. awamori* at 35 days

Parameters	CON	OP_10_	OA1	OA2	SEM	*p*-Value
**Immune organs**						
Thymus	0.121	0.118	0.128	0.122	0.007	0.093
Spleen	0.107	0.112	0.109	0.114	0.019	0.064
Bursa of Fabricius	0.134^b^	0.129^b^	0.142^b^	0.197^a^	0.002	< 0.001
**Immunoglobulin**						
IgG	415^c^	395^c^	486^b^	557^a^	41.27	0.021
IgA	252	246	255	264	16.08	0.085
IgM	127	131	138	133	12.44	0.116

CON, a basal diet; OP_10_, diet contains 10% olive pulp; OA1, diet contains 10% olive pulp with *A. awamori* at 100 mg; OA2, diet contains 10% olive pulp with *A. awamori* at 200 mg. a–b Means within the same row with different superscripts differ. SEM, standard error of means

### Cecum microbial enumeration

Regarding the cecum, heat stress and the addition of *A. awamori* showed a significant effect on the microbial content of the intestine, as shown in Table 9. Supplementing the diet with 200 mg *A. awamori* led to an increase in *Lactobacillus spp*. count, whilst the *E. coli* count slightly decreased (*P* < 0.05) in broiler fed AO2 compared with the other groups. In contrast, no significant effects (*P* < 0.05) were observed for *Enterococcus* and *coliform counts*. Cecum microbial content was not affected in the OP_10_ group (*P* < 0.05) compared with the control group under heat stress conditions.

**Table 9:** Cecum microbial enumeration (Log^10^ CFU g), of heat-stressed broilers fed diets containing olive pulp with different levels of *A. awamori* at 35 days

Parameters	CON	OP_10_	OA1	OA2	SEM	*p*-Value
Lactobacillus	5.06^b^	4.97^b^	5.24^b^	6.71^a^	0.251	0.010
Total Coliforms	4.43	4.21	4.35	4.51	0.676	0.224
Enterococcus	6.11	5.94	6.13	6.08	0.058	0.109
Escherichia coli	4.39^b^	4.46^b^	4.02^ab^	3.34^b^	0.034	< 0.001

CON, a basal diet; OP_10_, diet contains 10% olive pulp; OA1, diet contains 10% olive pulp with *A. awamori* at 100 mg; OA2, diet contains 10% olive pulp with *A. awamori* at 200 mg. a–b Means within the same row with different superscripts differ. SEM, standard error of means

## Discussion

Recently, nutritionists have been attracted by the uses of direct-fed microbials (*A. awamori*) in chicken feed for their beneficial effect on heat-stressed broiler performance by managing gut microbes, and enhancing the immune status of the host [4, 5, 7]. Furthermore, probiotics enhance the digestion of nutrients, especially in unconventional feed materials, by reducing anti-nutritive substances [5]. In the present study, *A. awamori* was added to the diet that includes OP to improve the nutritional value of OP and to partially replace it in a heat-stressed broiler diet. As we expected, our results showed a significant improvement in the performance of heat-stressed birds fed a diet that includes OP with *A. awamori*.

Our results showed that supplementing with 200 mg *A. awamori* improved broiler growth performance indicated by the increase in BWG and the decrease in FCR under heat-stress conditions, in addition to enhancing the nutritional value of the OP in the diet. These results agree with those of Saleh et al. [12]. Similarly, Yamamoto et al. [24] have noticed a significant improvement in BWG and FCR of broilers after feeding on diets containing *A. awamori*. The improvement in growth performance might be due to the reduction in the anti-nutritional factor of the feed and the improvement in gut health [5]. These improvements are due to the activation of several health-promoting bacteria, improving the intestinal epithelial cells structure, and selectively stimulating their growth, as well as, stimulating the immune system, which enhances the welfare and health of the chickens [25]. In addition, adding *A. awamori* to the diet containing OP leads to stimulating the activity of some enzymes (active amylase, glucoamylase, and protease) in the digestive system, leading to an improvement in the metabolism of energy, protein, and carbohydrates [13]. The noticeable improvement in the productive performance in our results may be due to the efficient role of *A. awamori* in improving the intestinal microbial environment by decreasing the pH value, reducing disease-causing microbes, and decreasing the levels of antinutrients in OP, which improves broiler health and food utilization efficiency [4, 7, 12, 17]. This supports our hypothesis that adding *A. awamori* leads to improving the nutritional value of OP, in addition to reducing the negative effect of heat stress on broilers.

Our results indicated an improvement in the carcass traits through the increase in dressing percentage and the decrease in the abdominal fat content in broiler fed a diet containing 200 mg *A. awamori* and OP. This agrees with Yamamoto et al. [24]., who reported that there was a significant increase in the carcass weight of broilers when fed diets containing *A. awamori*. This improvement may be due to the growth-promoting action produced by *A. awamori* [26], which stimulates protein synthesis in muscle tissue [27]. The growth of chickens is primarily regulated by genes of the somatotropic axis that have a significant role in muscle development and growth, such as insulin-like growth factor 1 (IGF1) and the growth hormone secretagogue receptor (GHSR). The IGF1 gene plays an important role in stimulating amino acid uptake, protein synthesis, and glucose uptake [28]. GHSR plays an essential role in food conversion rate, while the over-expression of the IGF1 gene in the muscle tissue leads to boosting muscle growth in chickens [29]. Some studies reported that the addition of *A. awamori* as a probiotic had a role in stimulating both IGF1 and GHSR [28, 29], which explains the improvement in carcass weight by enhancing muscle growth in the present experiment. However, our results showed a significant reduction in abdominal fat in groups fed a diet containing *A. awamori*. These results were similar to those of Abdullah et al. [30] and Elbaz et al. [2] who found a significant reduction in the abdominal fat content of the broilers fed probiotics. Navidshad et al. [31] found that chickens fed on diets including probiotics have significantly lower abdominal fat. This may be attributed to certain microflora that is present in the gut of chicks impairing the absorption of cholesterol and bile acid [32], which led to reducing abdominal fat. The decrease in abdominal fat when adding *A. awamori* is beneficial for meat quality and economically. The decrease in abdominal fat indicates the role that *A. awamori* plays in the distribution of fat in the carcass, which may improve the quality of the meat, as the fatty acids increase in the tissues contributes to the succulence, quality, and flavor of meat [33]. In addition to that, there is an economic benefit, as the decrease in abdominal fat (slaughterhouse waste) increases carcass weight [3]. Therefore, it can be concluded from the previous results that the addition of *A. awamori* had a major role in improving carcass characteristics.

With the high costs of feed ingredients (about 75–80% of the total production costs) and exposed birds to much stress, it was necessary to track the digestion and absorption of nutrients to determine the extent of benefits to the bird, in addition to the role of additives in improving the utilization of unconventional feed ingredients [2]. Therefore, probiotics (*A. awamori*) were used in the current study to enhance nutrient digestion and reduce anti-nutrients in OP by improving the status of gastrointestinal and microbial composition, as well as, mitigating the negative effect of heat stress. Thus, we evaluated the nutrient digestibility and the digestive enzyme activity in chickens fed a diet that included OP with the addition of 200 mg *A. awamori*. Our results showed a significant improvement in the digestion of crude protein, and crude fiber, in addition to an increase in the level of protease and amylase enzymes in chickens that received a diet containing *A. awamori*. This is consistent with several reports that have confirmed the stimulating effect of probiotic supplementation on nutrient and enzyme activity [25, 28]. However, some reports indicated that the digestion of nutrients was not affected in chickens fed probiotics [34]. The difference in the results of previous reports may be due to the composition of probiotics, i.e., the type of microbe added to the experimental diet and its concentration. It is possible that the improvement in nutrient digestion is due to improved gut health [11], inhibition of pathogenic bacteria and reduction of intestine pH, and stimulation of the secretion of digestive enzymes [28]. We concluded from the results of our study that adding *A. awamori* improved the digestion of nutrients and enhanced the activity of enzymes, indicating that *A. awamori* could be used in a diet containing OP, to enhance the nutrition value of OP, in addition to the important role in improving feed utilization during exposure to heat stress.

The results of the current study showed that adding only OP or with *A. awamori* had a significant effect on lipid metabolism, meanwhile, it did not affect serum protein concentration and liver enzymes (AST and ALT). Some previous studies agree that there was no effect of OP or *A. awamori* supplementation on liver activity [35, 36]. In this study, adding *A. awamori* or OP decreased serum cholesterol, while the triglyceride and LDL level decreased and HDL level increased in broiler fed 200 mg *A. awamori* compared to other groups. These results agree with those obtained by Kim et al. [37] and Elbaz et al. [5] who found a decrease in cholesterol and triglycerides levels in birds fed probiotics, which may be due to the inhibition of cholesterol biosynthesis. Hypocholesterolemia may be a consequence of feeding on olive pulp due to its high content of soluble fiber, which increases the viscosity of food, leading to decreasing intestinal motility, postponing the evacuation of the gut, and reducing fat absorption [38]. Likewise, It has been noted that *A. awamori* affects lipid metabolism, by inhibiting 3-hydroxyl-3- methylglutaryl-coenzyme (HMG-CoA) reductase that leads to cholesterol-lowering [12]. From our results, it can be concluded that the decrease in serum lipids resulted from the dual effect of adding OP and *A. awamori*.

Chick’s oxidation state is affected by many factors, the most important of which are environmental (including heat stress) and nutritional changes. More recently, the involvement of heat stress in inducing oxidative stress has received much interest. Oxidative stress is defined as the presence of reactive species over the available antioxidant capacity of poultry cells. Reactive species can modify several biologically cellular macromolecules and can interfere with cell signaling pathways. Furthermore, there has been an ever-increasing interest in the use of some feed additives that have potential antioxidant properties for poultry. Thus, the estimation of oxidative enzymes in the blood or tissues is a significant indicator that shows the extent of the bird’s exposure to oxidative stress. In the current study, the addition of *A. awamori.* and the partial replacement of the diet with OP led to a significant effect on the oxidative stress enzymes. MDA significantly decreased, while SOD and GPx significantly increased in groups that received 200 mg *A. awamori* compared to other groups. The decrease in MDA level indicates how the cell membrane is not affected by the oxidative stress of the bird with feed additives [35, 39]. The addition of *A. awamori*. improved the oxidative state of the bird exposed to heat stress in the current study. In agreement with our study, Saleh et al. [12]., indicated that probiotics have antioxidant properties. Moreover, the present study showed a positive result in the antioxidant status when adding OP. The positive effect of OP on broilers’ antioxidant status may be due to polyphenols (oleuropein and hydroxytyrosol) in OP, that boost the antioxidant activity of birds [4, 40]. It can be concluded that adding olives with *A. awamori* is considered a protective mechanism against heat stress to mitigate the oxidative stress of broilers.

In this study, we examined whether supplementing *A. awamori* will enhance broiler chickens’ immune responses. Immunoglobulins and lymphoid organic and their responses were used as an efficacy indicator of that supplementation. The current study showed that the immune response increased significantly regarding the IgG level and relative weight bursa of Fabricius, as a result of the addition of 200 mg *A. awamori* in broiler diets under heat stress. The same results were reported by Humam et al. [41]and Yin et al. [42], where feeding probiotics had a positive impact on immune response, indicated by the increase in the serum levels of IgA and IgG in broilers. In addition, Qiu et al. [43] reported that broilers fed diets containing probiotics exhibited a rapid production rate in serum IgG compared with a control diet. It has been proven that the increase in the relative weight of the immune organs through food additives is usually associated with better immunity [21]. This is consistent with our results, which indicated the improvement in the immune response (by the relative weight of the bursa of Fabricius) of birds fed *A. awamori.* under heat stress. A. awamori., as a probiotic, has a modulating impact on gut microbes and plays a beneficial role in the development of the immune system [21, 42]. The noticeable amelioration in immunity may be due to the role of *A. awamori.* as an antimicrobial, and anti-inflammatory [43], which helps in providing nutrients necessary for the development of the immune system by promoting the proliferation of lymphocytes in the primary immune organs and enhancing intestinal integrity [44], thus stimulating immune response, and produces immunoglobulins. From that can be deduced, adding *A. awamori*. had a significant role in enhancing immune response.

Dietary compositional differences, diet physical properties, and the presence of feed additives have a significant role in altering broiler gut microbial composition and functionality [45]. Furthermore, gut microbes, play an important role in utilizing food by stimulating the secretion of some enzymes, digestion and absorption of nutrients, and enhancing immunity [46]. The results of the current study showed a positive effect of adding 200 mg *A. awamori* on the cecum population in heat-stressed broiler chickens by increasing the *Lactobacillus* population and decreasing the *E. coli* population. Numerous reports indicated that physiological effects related to probiotics have been proven to boost the growth of *Lactobacillus* and inhibit *E. coli* in broiler gut [46, 47] which is consistent with our results. Adding probiotics provides a facilely available substrate for the gut-beneficial microbial to grow and reduce toxic microbial activities [48], which promotes birds’ health during heat stress. Our results indicated that *A. awamori* addition altered the gut microbiota diversity, which is beneficial to bird health. From the above, we conclude that adding probiotics had a significant role in improving the utilization of agricultural waste and reducing the burden resulting from heat stress by improving gut microbial content and enhancing the utilization of the diet [2, 49]. Similarly, our results indicated that adding *A. awamori* (as a probiotic) to a diet containing OP had beneficial effects on birds’ health under heat-stress conditions, in addition to improving the nutritional value of OP.

## Conclusions

The current study revealed that the addition of 200 mg *A. awamori.* had a role in improving the nutritional value of OP, which supports its use with a mixture of *A. awamori.* as a partial replacement in the broiler diet. Performance, serum lipid profile, antioxidant functions, immune responses, and intestinal microbiota diversity of heat-stressed broilers improved when fed *A. awamori*. It can be concluded that adding *A. awamori* enhanced the nutritional value of OP while reducing the negative effects of heat-stress in broilers.

## Data Availability

The datasets used and/or analysed during the current study are available from the corresponding author upon reasonable request.

## Abbreviations

A. awamori: *Aspergillus awamori*
ALP: Alkaline phosphatase
ALT: Alanine aminotransferase
AST: Aspartate aminotransferase
BWG: Body weight gain
CON: Chicks fed a basal diet
E. coli: Escherichia coli
FCR: Feed conversion ratio
FI: Feed intake
GPx: Glutathione peroxidase
HDL: High-density lipoprotein
IgA: Immunoglobulins A
IgG: Immunoglobulins G
IgM: Immunoglobulins M
LBW: Live body weight
LDL: Low-density lipoprotein
MDA: Malondialdehyde
OA1: Chicks fed a diet containing OP with A. awamori at 100 mg per kg
OA2: Chicks fed a diet containing OP with A. awamori at 200 mg per kg
OP: Chicks fed a diet containing 10% OP
SOD: Superoxide dismutase

## References

[1] Abd El-Moneim AE, Sabic EM, Abu-Taleb AM (2022). Influence of dietary supplementation of irradiated or non-irradiated olive pulp on biochemical profile, antioxidant status and immune response of Japanese quails. Biol Rhythm Res.

[2] Elbaz AM (2021). Effects of Diet containing fermented Canola Meal on performance, blood parameters, and Gut Health of Broiler Chickens. J World’s Poult Res.

[3] Elbaz AM, Zaki EF, Morsy AS (2022). Productive performance, physiological responses, carcass traits, and meat quality of broiler chickens fed quinoa seeds. Adv Anim Vet Sci.

[4] Al-Harthi MA. The effect of different dietary contents of olive cake with or without Saccharomyces cerevisiae on egg production and quality, inner organs and blood constituents of commercial layers. Eur Poult Sci. 2015;79. 10.1399/eps.2015.83.

[5] Elbaz AM, Thabet HA, Gad GG (2020). Productive and physiological performance of broilers fed diets containing different levels of olive pulp. J Anim Poult Prod.

[6] Kalogeropoulos N, Tsimidou MZ (2014). Antioxidants in Greek virgin olive oils. Antioxidants.

[7] Saleh AA, Paray BA, Dawood MA (2020). Olive cake meal and Bacillus licheniformis impacted the growth performance, muscle fatty acid content, and health status of broiler chickens. Animals.

[8] Abdel-Moneim AME, Shehata AM, Khidr RE, Paswan VK, Ibrahim NS, El-Ghoul AA, Aldhumri SA, Gabr SA, Mesalam NM, Elbaz AM, Elsayed MA (2021). Nutritional manipulation to combat heat stress in poultry–A comprehensive review. J Therm Biol.

[9] Abdel-Moneim AME, Shehata AM, Mohamed NG, Elbaz AM, Ibrahim NS (2022). Synergistic effect of Spirulina platensis and selenium nanoparticles on growth performance, serum metabolites, immune responses, and antioxidant capacity of heat-stressed broiler chickens. Biol Trace Elem Res.

[10] Wang WC, Yan FF, Hu JY, Amen OA, Cheng HW (2018). Supplementation of Bacillus subtilis-based probiotic reduces heat stress-related behaviors and inflammatory response in broiler chickens. J Anim Sci.

[11] Elbaz AM, Ibrahim NS, Shehata AM, Mohamed NG, Abdel-Moneim AME (2021). Impact of multi-strain probiotic, citric acid, garlic powder or their combinations on performance, ileal histomorphometry, microbial enumeration and humoral immunity of broiler chickens. Trop Anim Health Prod.

[12] Saleh AA, Eid YZ, Ebeid TA, Kamizono T, Ohtsuka A, Hayashi K (2011). Effects of feeding aspergillus awamori and aspergillus Niger on growth performance and meat quality in broiler chickens. J Poult Sci.

[13] Gracia MI, Aranibar M, Lazaro R, Medel P, Mateos GG (2003). Alpha-amylase supplementation of broiler diets based on corn. Poult Sci.

[14] Chowdhury R, Islam KMS, Khan MJ, Karim MR, Haque MN, Khatun M, Pesti GM (2009). Effect of citric acid, avilamycin, and their combination on the performance, tibia ash, and immune status of broilers. Poult Sci.

[15] AOAC BA. Association of official analytical chemists. Official Methods Anal. 1990;12.

[16] NRC. National Research Council. Nutrient Requirements of Poultry 9th ed. Composition of poultry feedstuffs. National Academy Press, Washington, DC, USA. p.p. 1994;61–75.

[17] Elbaz AM, Farrag B, Mesalam NM, Basuony HA, Badran AM, Abdel-Moneim AM (2023). Growth performance, digestive function, thyroid activity, and immunity of growing rabbits fed olive cake with or without Saccharomyces cerevisiae or citric acid. Trop Anim Health Prod.

[18] Gaithersburg, AOAC (2003). Official methods of analysis of AOAC.

[19] Najafi MF, Deobagkar DN, Mehrvarz M, Deobagkar DD (2006). Enzymatic properties of a novel highly active and chelator resistant protease from a Pseudomonas aeruginosa PD100. Enzym Microb Technol.

[20] Lynn KR, Clevette-Radford NA (1984). Purification and characterization of hevain, a serine protease from Hevea brasiliensis. Phytochemistry.

[21] Elbaz AM, Ashmawy ES, Salama AA, Abdel-Moneim AME, Badri FB, Thabet HA (2022). Effects of garlic and lemon essential oils on performance, digestibility, plasma metabolite, and intestinal health in broilers under environmental heat stress. BMC Vet Res.

[22] Czerwiński J, Højberg O, Smulikowska S, Engberg RM, Mieczkowska A (2012). Effects of sodium butyrate and salinomycin upon intestinal microbiota, mucosal morphology and performance of broiler chickens. Arch Anim Nutr.

[23] Duncan DB (1955). Multiple ranges and multiple F tests. Biometrics.

[24] Yamamoto M, Saleh F, Tahir M, Ohtsuka A, Hayashi K (2007). The effect of Koji-feed (fermented distillery by-product) on the growth performance and nutrient metabolizability in broiler. J Poult Sci.

[25] Saleh AA, Hayashi K, Ijiri D, Ohtsuka A (2014). Beneficial effects of aspergillus awamori in broiler nutrition. World’s Poult Sci J.

[26] Kamizono T, Nakashima K, Ohtsuka A, Hayashi K, Bioscience (2010). Biotechnol Biochem.

[27] Chang G, Liu X, Liao J, Chen R, Luan D, Zhang Y, Ma T, Zhou W, Wang K, Chen G (2011). Temporal and spatial expression of the pax-7 gene during chickenembryo and postnatal development. J Anim Veterinary Adv.

[28] Saleh AA, Amber K, El-Magd MA, Atta MS, Mohammed AA, Ragab MM, El-Kader A (2014). Integrative effects of feeding aspergillus awamori and fructooligosaccharide on growth performance and digestibility in broilers: Promotion muscle protein metabolism. Biomed Res Int.

[29] Mitchell PJ, Johnson SE, Hannon K (2002). Insulin-like growth factor I stimulates myoblast expansion and myofiber development in the limb. Dev Dynamics: Official Publication Am Association Anatomists.

[30] Abdullah N, Jalaludin S, Wong MC, Ho YW (2006). Effects of Lactobacillus feed supplementation on cholesterol, fat content and fatty acid composition of the liver, muscle and carcass of broiler chickens. Anim Res.

[31] Navidshad B, Adibmoradi M, Pirsaraei ZA (2016). Effects of dietary supplementation of aspergillus originated prebiotic (Fermacto) on performance and small intestinal morphology of broiler chickens fed diluted diets. Italian J Anim Sci.

[32] Li G (2012). Intestinal probiotics: interactions with bile salts and reduction of cholesterol. Procedia Environ Sci.

[33] Lippens M. The influence of feed control on the growth pattern and production parameters of broiler chickens (Doctoral dissertation, Ghent University). 2003. http://hdl.handle.net/1854/LU-521843.

[34] Zhang ZF, Kim IH (2014). Effects of multistrain probiotics on growth performance, apparent ileal nutrient digestibility, blood characteristics, cecal microbial shedding, and excreta odor contents in broilers. Poult Sci.

[35] Abd El-Moneim AE, Sabic EM (2019). Beneficial effect of feeding olive pulp and aspergillus awamori on productive performance, egg quality, serum/yolk cholesterol and oxidative status in laying Japanese quails. J Anim Feed Sci.

[36] Saleh AA, Gálik B, Arpášová H, Capcarová M, Kalafová A, Šimko M, Juráček M, Rolinec M, Bíro D, Abudabos A (2017). Synergistic effect of feeding aspergillus awamori and lactic acid bacteria on performance, egg traits, egg yolk cholesterol, and fatty acid profile in laying hens. Italian J Anim Sci.

[37] Kim SS, Galaz GB, Pham MA, Jang JW, Oh DH, Yeo IK, Lee KJ (2009). Effects of dietary supplementation of a meju, fermented soybean meal, and aspergillus oryzae for juvenile parrot fish (Oplegnathus fasciatus). Asian-Australasian J Anim Sci.

[38] Razdan A, Pettersson D (1994). Effect of chitin and chitosan on nutrient digestibility and plasma lipid concentrations in broiler chickens. Br J Nutr.

[39] Yuan B, Ohyama K, Bessho T, Uchide N, Toyoda H (2008). Imbalance between ROS production and elimination results in apoptosis induction in primary smooth chorion trophoblast cells prepared from human fetal membrane tissues. Life Sci.

[40] Bulotta S, Celano M, Lepore SM, Montalcini T, Pujia A, Russo D (2014). Beneficial effects of the olive oil phenolic components oleuropein and hydroxytyrosol: focus on protection against cardiovascular and metabolic diseases. J Translational Med.

[41] Humam AM, Loh TC, Foo HL, Samsudin AA, Mustapha NM, Zulkifli I, Izuddin WI. Effects of feeding different postbiotics produced by Lactobacillus plantarum on growth performance, carcass yield, intestinal morphology, gut microbiota composition, immune status, and growth gene expression in broilers under heat stress. Animals. 2019;9, p.644. 10.3390/ani9090644. 10.3390/ani9090644 PMC6770893 31480791

[42] Yin YL, Tang ZR, Sun ZH, Liu ZQ, Li TJ, Huang RL, Ruan Z, Deng ZY, Gao B, Chen LX, Wu GY (2008). Effect of galacto-mannan-oligosaccharides or chitosan supplementation on cytoimmunity and humoral immunity in early-weaned piglets. Asian-Australasian J Anim Sci.

[43] Qiu R, Croom J, Ali RA, Ballou AL, Smith C, Ashwell CM, Hassan HM, Chiang CC, Koci MD (2012). Direct fed microbial supplementation repartitions host energy to the immune system. J Anim Sci.

[44] Kareem KY, Loh TC, Foo HL, Asmara SA, Akit H (2017). Influence of Postbiotic RG14 and inulin combination on Caecal Microbiota, Organic Acid concentration and cytokine expression in broiler chickens. Poult Sci.

[45] Abdel-Moneim AME, Elbaz AM, Khidr RES, Badri FB (2020). Effect of in ovo inoculation of Bifidobacterium spp. on growth performance, thyroid activity, ileum histomorphometry, and microbial enumeration of broilers. Probiotics Antimicrob Proteins.

[46] Zhao Y, Zeng Y, Zeng D, Wang H, Sun N, Xin J, Zhou M, Yang H, Lei L, Ling H, Khalique A. Dietary probiotic supplementation suppresses subclinical necrotic enteritis in broiler chickens in a microbiota-dependent manner. Front Immunol. 2022;13. 10.3389/fimmu.2022.855426. 10.3389/fimmu.2022.855426 PMC8972058 35371037

[47] Oyeagu CE, Mlambo V, Muchenje V, Marume U (2019). Effect of dietary supplementation of aspergillus xylanase on broiler chickens performance. Iran J Appl Anim Sci.

[48] ALbazaz R, BAL EB (2014). Microflora of digestive tract in poultry. KSÜ Doğa Bilimleri Dergisi.

[49] Sohail MU, Hume ME, Byrd JA, Nisbet DJ, Ijaz A, Sohail A, Shabbir MZ, Rehman H (2012). Effect of supplementation of prebiotic mannan-oligosaccharides and probiotic mixture on growth performance of broilers subjected to chronic heat stress. Poult Sci.

